# Combination of Bacteriophages and Antibiotics for Prevention of Vascular Graft Infections—An In Vitro Study

**DOI:** 10.3390/ph16050744

**Published:** 2023-05-13

**Authors:** Stefan Ruemke, Evgenii Rubalskii, Christina Salmoukas, Kristina Hermes, Ruslan Natanov, Tim Kaufeld, Oleksandr Gryshkov, Vitalii Mutsenko, Maxim Rubalsky, Karin Burgwitz, Birgit Glasmacher, Axel Haverich, Saad Rustum, Christian Kuehn

**Affiliations:** 1 Department of Cardiac, Thoracic, Transplantation and Vascular Surgery, Hannover Medical School, 30625 Hannover, Germany; 2 Lower Saxony Centre for Biomedical Engineering, Implant Research and Development, 30625 Hannover, Germany; 3 Institute for Multiphase Processes, Leibniz University Hannover, 30823 Garbsen, Germany; 4 Department of Microbiology and Virology, Astrakhan State Medical University, 414000 Astrakhan, Russia

**Keywords:** implant-associated infection, vascular graft, prevention, bacteriophage, antibiotics, fibrin glue, coating

## Abstract

(1) Background: Implant-associated bacterial infections are usually hard to treat conservatively due to the resistance and tolerance of the pathogens to conventional antimicrobial therapy. Bacterial colonization of vascular grafts may lead to life-threatening conditions such as sepsis. The objective of this study is to evaluate whether conventional antibiotics and bacteriophages can reliably prevent the bacterial colonization of vascular grafts. (2) Methods: Gram-positive and Gram-negative bacterial infections were simulated on samples of woven PET gelatin-impregnated grafts using *Staphylococcus aureus* and *Escherichia coli* strains, respectively. The ability to prevent colonization was evaluated for a mixture of broad-spectrum antibiotics, for strictly lytic species-specific bacteriophage strains, and for a combination of both. All the antimicrobial agents were conventionally tested in order to prove the sensitivity of the used bacterial strains. Furthermore, the substances were used in a liquid form or in combination with a fibrin glue. (3) Results: Despite their strictly lytic nature, the application of bacteriophages alone was not enough to protect the graft samples from both bacteria. The singular application of antibiotics, both with and without fibrin glue, showed a protective effect against *S. aureus* (0 CFU/cm^2^), but was not sufficient against *E. coli* without fibrin glue (M = 7.18 × 10^4^ CFU/cm^2^). In contrast, the application of a combination of antibiotics and phages showed complete eradication of both bacteria after a single inoculation. The fibrin glue hydrogel provided an increased protection against repetitive exposure to *S. aureus* (*p* = 0.05). (4) Conclusions: The application of antibacterial combinations of antibiotics and bacteriophages is an effective approach to the prevention of bacteria-induced vascular graft infections in clinical settings.

## 1. Introduction

Due to an ageing population and the first-world lifestyle, the number of cardiovascular disease cases requiring surgery is increasing. In these cases, industrially manufactured prosthetic materials are often used. In the context of these surgical procedures, there is also a risk of implant-associated infections. Preventive measures are therefore of great importance. In addition to systemic perioperative antibiotic use, the impregnation of the prosthesis material with a suitable antibiotic has been shown to be effective [1].

The development of chronic prosthetic infections generally requires the removal of the infected implant, followed by prolonged antibacterial therapy and eventually the implantation of a new medical device. Currently, a combination of gentamicin and vancomycin is often used for a topical antibacterial effect in cardiovascular and orthopedic surgery [2,3]. However, in the field of cardiovascular surgery, prolonged absence of the prosthesis is not an option. In these circumstances, a newly implanted graft carries a high risk of recurrent infection, which is associated with high mortality.

The failure of antibiotic therapy against recurrent graft infection is associated with biofilm formation on the surface of the prosthesis [4]. Biofilms are known to promote the formation of a viable but nonculturable state or persister cells, which are tolerant to high doses of antibiotics [5].

Bacteriophages are viruses that can specifically infect bacteria without affecting eukaryotic cells. Typically, phages infect bacteria within a species that serves as their host and use its metabolism to replicate. Eventually, the bacterial cell wall is lysed by a phage-produced endolysin, and new phages are released [6]. Obligatory lytic bacteriophages are known to overcome the failures of conventional antibacterial therapy and to show high efficacy and safety in personalized phage therapy settings [7,8]. The target bacteria must be identified in, for example, conventional blood cultures or samples from punctures of the infected site prior to the treatment. However, bacteria have a number of mechanisms that allow them to evade phage infection, reduce its efficiency, and create conditions for phage–bacteria coexistence [9,10]. Therefore, the search for strategies to evade these mechanisms and increase the efficiency of phage therapy and phage prophylaxis is promising.

Earlier studies showed the beneficial effect of the joint use of some antibiotics and phages [11,12]. For that reason, a combination of phages and antibiotics might be a promising approach to the prevention of vascular graft infections.

In addition, a suitable drug delivery system is required for the prolonged local application of antibacterial drugs. Fibrin glue is a hydrogel sealant consisting of two components, fibrinogen and thrombin, and it has been widely used in surgical practice. Previous studies have shown that the incorporation of antibiotics into fibrin glue is a safe, effective, and clinically relevant method of drug delivery in cardiovascular surgery [13,14]. Fibrin glue has also been shown to be an effective tool for the prolonged drug delivery of bacteriophages [15].

In this in vitro study, we compared the efficacy of topical antibiotics, bacteriophages, and their combination against two of the most common bacteria involved in vascular graft infections. The substances were tested on a vascular graft by impregnation of the graft or in a mixture with a fibrin glue coating.

## 2. Results

### 2.1. Surface Coating of the Grafts

**Figure 1:**
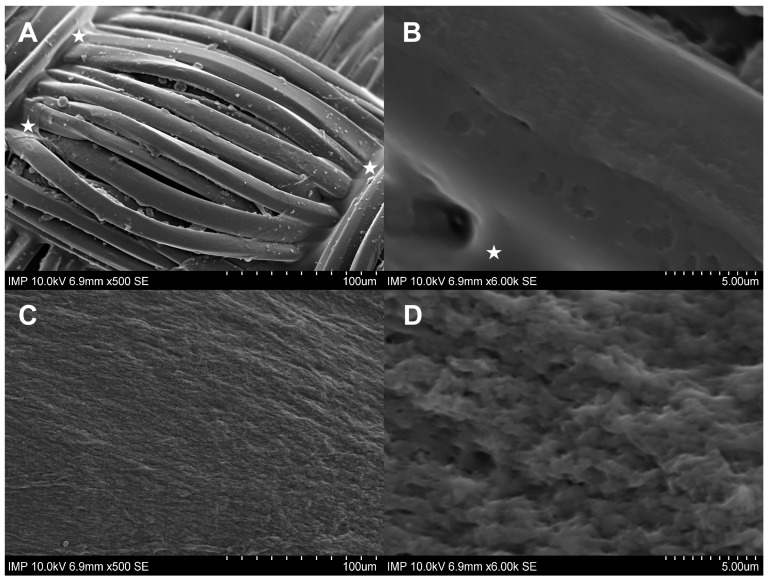
SEM images of the negative control samples without (**A**,**B**) and with fibrin glue coating (**C**,**D**). The woven structure and the gelatin-sealed (★) surface of the prosthesis can be recognized in **A**,**B**. In images **C**,**D**, the surface structure changed into a continuous, rough fibrin glue layer.

Gelweave™ is a gelatin-sealed woven PET vascular graft. Initially designed to prevent intraoperative diffuse bleeding under physiological conditions, the gelatin coating did not show complete continence after prolonged overnight incubation in intact TSB solution. In comparison, the fibrin glue coating remained constant, as shown by scanning electron microscopy (SEM) imaging (Figure 1).

### 2.2. Antibacterial Loads of Graft Samples

#### 2.2.1. Uncoated Grafts

The measured volumes of antibacterial substance uptake showed no statistically significant differences between the five tested subgroups (Supplementary Materials, Table S1 and Figure S1). The calculated final amounts of eluted antibiotics from the impregnated graft samples showed robust inhibitory concentrations (all samples > 6 µg/mL) for vancomycin, but not for gentamicin (11/15 samples < 4 µg/mL) (Supplementary Materials, Table S2). The calculated amount of eluted phages showed a statistically significant higher titer for the phage NZPT-SA7 than the values measured after 1 h of incubation. For the samples containing phage ECD7, no significant differences between the calculated and the measured phage titer were found (Supplementary Materials, Table S3 and Figure S2).

#### 2.2.2. Coated Grafts

The developed procedure of fibrin glue application was sufficient to provide a full coating and mechanical protection of the graft samples with a standardized volume of antibacterial substances (Supplementary Materials, Figure S3). The values measured for the released PFUs within the first hour were 2.16 × 10^6^ and 6.44 × 10^6^ per sample for the NZPT-SA7 and ECD7, respectively.

### 2.3. Antibacterial Activity against Single Contamination

#### 2.3.1. Activity against *Staphylococcus aureus*

**Figure 2:**
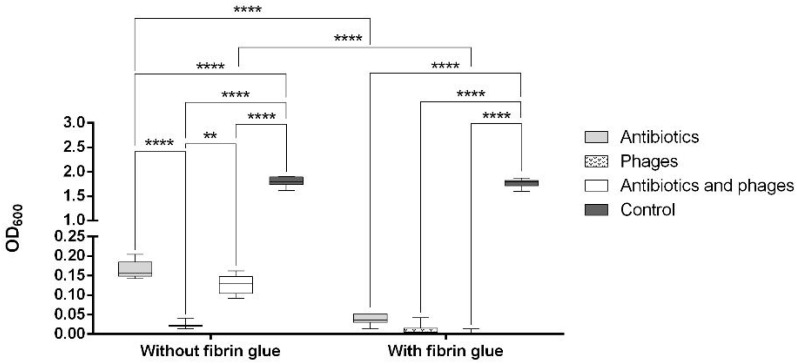
Value of OD_600_ of the *S. aureus* suspension after co-incubation with the graft samples (**: *p* < 0.01; ****: *p* < 0.0001).

The OD_600_ of the staphylococcal suspension was significantly decreased (*p* < 0.0001) in all the treated graft samples compared to the untreated positive controls, both with and without fibrin coating (Figure 2). Moreover, the antibiotics, by themselves as well as in a combination with bacteriophages, showed a significantly higher antibacterial efficacy in the fibrin-coated samples (*p* < 0.0001).

**Figure 3:**
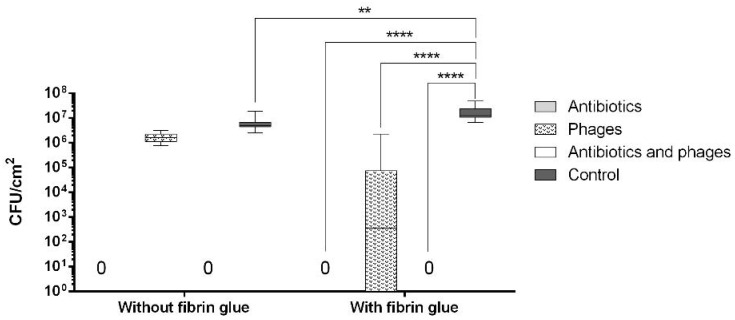
Count of CFU on the surface of the graft samples after co-incubation with *S. aureus* (**: *p* < 0.01; ****: *p* < 0.0001).

**Figure 4:**
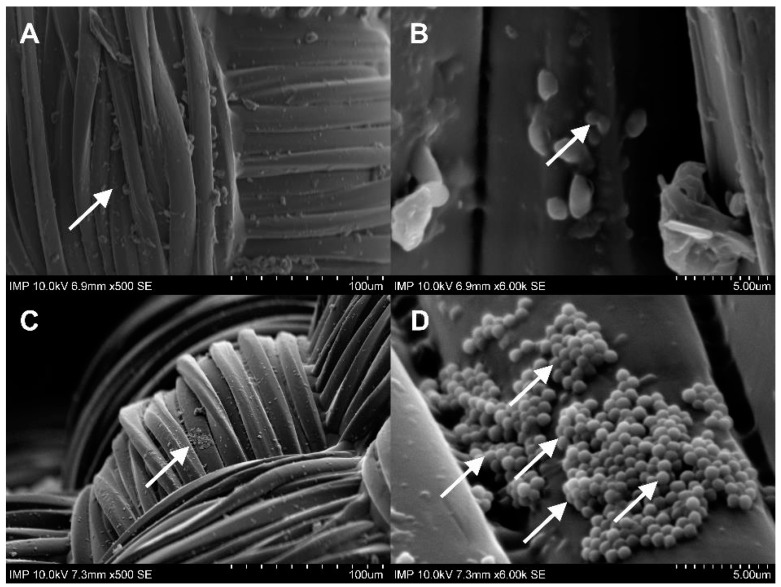
SEM images of graft samples with antibiotics and phages (**A**,**B**) and control samples (**C**,**D**) without fibrin coating after co-incubation with *S. aureus*. White arrows point to *S. aureus*.

**Figure 5:**
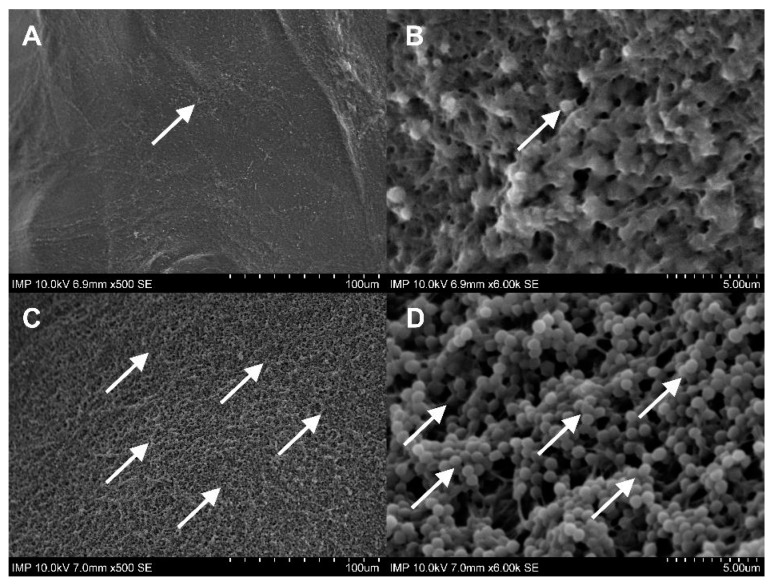
SEM images of graft samples with antibiotics and phages (**A**,**B**) and control samples (**C**,**D**) with fibrin coating after co-incubation with *S. aureus*. White arrows point to *S. aureus*.

As can be seen in Figure 3, both the coated and the uncoated samples containing antibiotics and antibiotics with phages were free from microbiologically detectable bacteria attached to their surface. A statistically significant difference from the control group was only observed in the samples with fibrin glue (*p* < 0.0001). We observed a significantly increased bacterial burden of the fibrin-coated control samples compared to the uncoated ones. This finding was confirmed by the following SEM images (Figure 4C,D and Figure 5C,D).

#### 2.3.2. Activity against *Escherichia coli*

**Figure 6:**
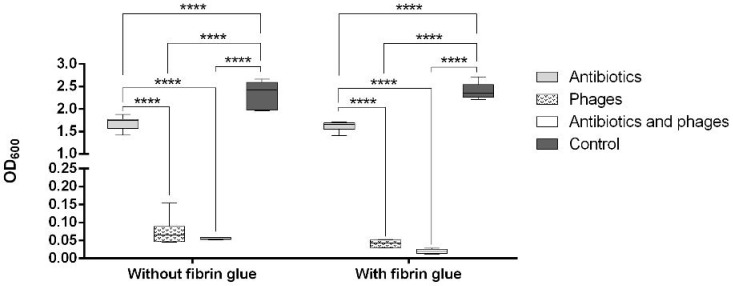
Value of OD_600_ of the *E. coli* suspension after co-incubation with the graft samples (****: *p* < 0.0001).

The *E. coli* suspension showed significantly lower OD_600_ values in all the antibacterial-treated samples compared to the controls (*p* < 0.0001). Moreover, all the phage-containing samples showed a significantly decreased OD_600_ (*p* < 0.0001) compared to the samples with antibiotics alone (Figure 6).

**Figure 7:**
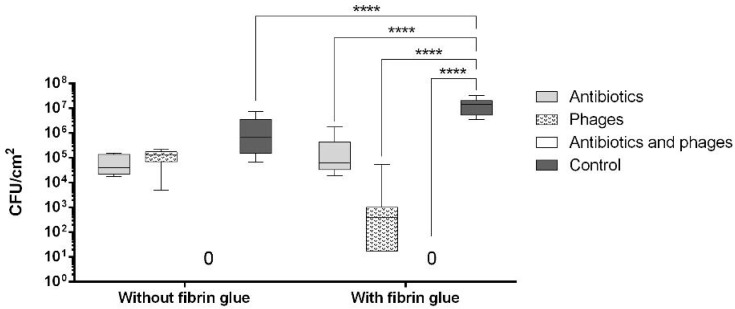
Count of CFUs on a surface of the graft samples after co-incubation with *E. coli* (****: *p* < 0.0001).

**Figure 8:**
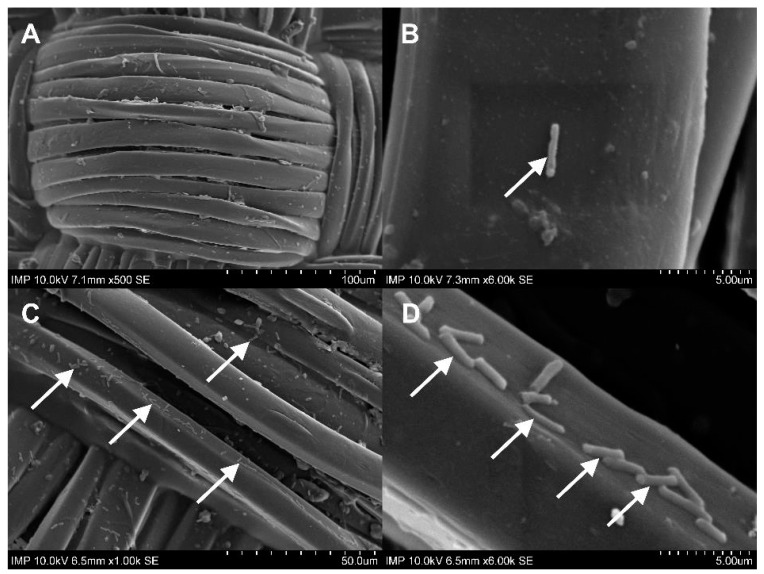
SEM images of graft samples with antibiotics and phages (**A**,**B**) and control samples (**C**,**D**) without fibrin coating after co-incubation with *E. coli*. White arrows point to *E. coli*.

**Figure 9:**
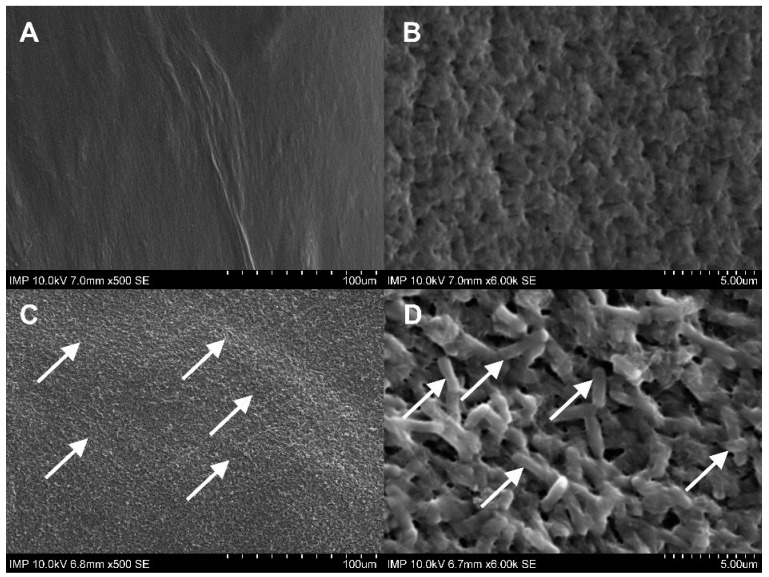
SEM images of graft samples with antibiotics and phages (**A**,**B**) and control samples (**C**,**D**) with fibrin coating after co-incubation with *E. coli*. White arrows point to *E. coli*.

After contamination with the *E. coli* suspension, both the fibrin-coated and the uncoated samples containing antibiotics with phages were free of microbiologically detectable bacteria attached to their surface (Figure 7). There was no statistically significant difference in the OD_600_ of the *E. coli* suspension between the coated and uncoated sample types in the same experimental groups. However, within the same sample type, a statistically significant difference was observed in all cases except for the phage and the phage–antibiotic groups. Low optical density values were observed in these four groups (Figure 6). The increase in colony-forming unit (CFU) counts in the fibrin-coated control samples was similarly confirmed by SEM imaging (Figure 8C,D and Figure 9C,D; Supplementary Materials, Figures S4 and S5).

### 2.4. Antibacterial Activity against Recurrent Contamination

**Figure 10:**
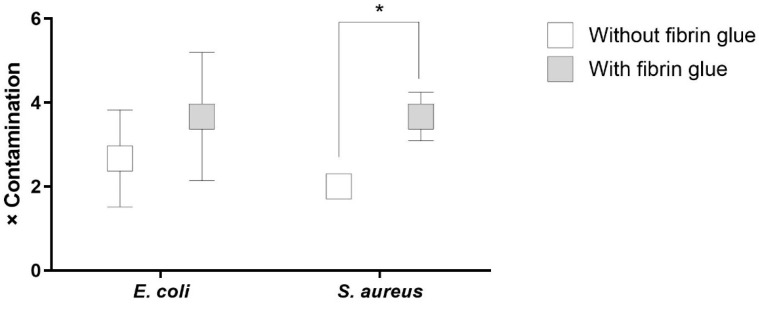
Contamination frequency of samples treated with a combination of antibiotics and phages providing the first microbiologically detectable burden (*: *p* = 0.05).

The antibacterial activity against repetitive contamination was assessed for the samples containing a combination of antibiotics and phages in order to estimate the potential time and reliability of the antibacterial action in terms of the severity of the burden caused by *S. aureus* and *E. coli*. The results demonstrated a better prophylactic potency of the fibrin glue-coated grafts compared to the uncoated ones (Supplementary Materials, Table S4). This tendency was statistically significant in the experiment with *S. aureus* (Figure 10).

## 3. Discussion

In the field of cardiac and vascular surgery, the prevention of infection and reinfection of vascular grafts is of fundamental importance. The consequences for the patients of such postoperative complications are serious [16]. The spectrum of complications is diverse and ranges from impaired wound healing and vascular wall erosion to fulminant sepsis. The aim of the presented work was to investigate the most promising clinical approach for the local prevention of bacterial vascular-graft-associated infections, including the combined use of antibiotics and bacteriophages.

Gram-positive pathogens such as *S. aureus* and Gram-negative bacteria such as *E. coli* are common causes of prosthesis-associated infections [17]. Therefore, the well-known combination of the antibiotics vancomycin and gentamicin was used in the present work [2,3]. The most suitable bacteriophages and their necessary concentrations were determined in an in vitro test series by utilizing spot tests and plaque assays on the used bacterial strains. The bacteriophage strains NZPT-SA7 and ECD7, used against *S. aureus* and *E. coli*, respectively, were chosen after showing the most promising results in the preliminary phagogram testing; they are also known for having a broad host range of representatives of the genus *Kayvirus* and the used coliphage [18,19].

Gelatin-sealed vascular grafts are already standard in aortic surgery. Gelatin hydrogel is used to prevent possible blood leakage from the prosthesis. At the same time, within 14 days after implantation, the biomass of the sealant resorbs [20]. This biodegradation profile makes the gelatin matrix of vascular prostheses a promising depot for antibacterial substances. According to the results of our studies with control samples, residual gelatin was observed predominantly in the contact areas of the transverse fibers of the graft. Most of the outer layer of the fibers remained uncovered by gelatin. However, a number of earlier studies showed that gelatin may play a role in protecting the implant surface from bacterial biofilm formation [2,20]. Therefore, further experiments have included groups with native gelatin-sealed grafts.

In view of the potentially limited volume of antimicrobial solution that can be absorbed by the vascular graft wall, the search for versatile carrier substances is necessary. Such carriers can, on the one hand, provide a depot function, ensuring a smooth, prolonged release of the antimicrobial agent into the surrounding tissues, and on the other hand, prevent premature washout of the active antimicrobial agent from the vascular prosthesis wall. Indeed, our in vitro results show that the concentration of gentamicin in the antibiotic mixture commonly used in vascular surgery may not always provide a robust inhibitory effect when the antibiotic is eluting into the surrounding tissue. The risk of an insufficient local concentration of antibacterial agents increases many times under real-life conditions because, during major vascular surgery, for example, large amounts of blood often wash around the vascular prosthesis, not only from the inside but also from the outside.

Our results on the example of the staphylococcal phage NZPT-SA7 and the coliphage ECD7 show that the dynamics of their elution from the wall of the vascular graft per time unit can differ dramatically, which also confirms the importance of carrier substances with a universally stable phage release profile. The combination of fibrin glue and antibiotics is well known and has been commonly used in surgery for years [21]. The additional intraoperative coating of vascular grafts with antibiotics has already become part of everyday clinical practice [1]. As earlier studies have shown, it is also possible to combine bacteriophages with fibrin glue [15] for a prolonged drug release. Studies performed on the staphylococcal phage NZPT-SA7 and the coliphage ECD7 showed that the number of phages released from the fibrin scaffold during the first hour was 1/35 and 1/12 of the initial amount, respectively. In comparison, the previously studied PA5 bacteriophage showed an average release of 1/4 of the number of virions within the first hour. It should be noted that the concentration of the bacteriophage PA5 in the earlier study was at least 10× higher than the bacteriophages NZPT-SA7 and ECD7 in the present work. Therefore, further detailed studies of the release profiles from the fibrin sealant of the most commonly used bacteriophages in clinical practice, with different concentrations and morphology, are necessary.

There are a number of case reports showing the great potency of phage therapy in cases of vascular graft-associated infections [7,22,23,24]. However, these clinical cases describe the combined use of bacteriophages and antibiotics, which is ethically justified but may call into question the effectiveness of bacteriophages. Therefore, the present study had a particular focus on the evaluation of the role of bacteriophages, antibiotics, and their combined use in a model experiment.

The promising synergistic effects that result from a combination of phages and antibiotics [11,12,25,26,27] led to the consideration of their use in the therapy/prevention of prosthetic graft infections with fibrin glue as a carrier substance. However, recent reports have shown that a combination of phages and antibiotics does not necessarily provide synergistic effects [28,29,30]. Moreover, some studies describe cases of phage–antibiotic antagonism [31,32,33,34,35]. Under these circumstances, the influence of the antibiotics on phages, as well as the influence of the phages on antibiotics, is necessary in order to define an optimal therapy plan for an individual patient. In addition, further standardized robust and reasonable methods are required to develop general recommendations for combined therapies. At the same time, the combination of phage–antibiotic systems containing more than two active substances remains understudied.

Taking into account the natural resistance of Gram-negative bacteria to vancomycin, our results show that the combination of phages and gentamicin works synergistically for the prevention of *E. coli* adhesion to a surface, but not for the inhibition of the planktonic bacteria growth. Gentamicin is an aminoglycoside antibiotic with a dose-dependent bactericidal activity. Firstly, the antibacterial action of aminoglycosides occurs due to the inhibition of ribosomal protein synthesis by the following mechanisms: the miscoding of mRNA [36]; the inhibition of mRNA and tRNA translocation [37,38]; and the inhibition of ribosome recycling [39] after binding to both 30S and 50S ribosomal subunits [40]. The antagonization of bacteriophage proliferation was shown experimentally for kanamycin [41]. Moreover, it was recently shown by Kever et al. that aminoglycosides are able to inhibit phage infection by the blocking of the phage life cycle during or after DNA injection but before DNA replication [41]. However, the synergistic action of bacteriophages and gentamicin was also reported [42,43].

Although we did not observe any statistical significance, the volume of uptake for phage NZPT-SA7 had a tendency to show a higher value than those of the other groups of uncoated samples (Supplementary Materials, Table S1 and Figure S1). Despite this, the titer of the released (and most likely absorbed) phage NZPT-SA7 in the uncoated samples (both with and without antibiotics) was significantly lower than calculated. This resulted in a lower level of multiplicity of infection (MOI) and greater chances for bacteria to escape the phage infection, which was confirmed by the high *S. aureus* burden for the phage-only group, but not for the group with antibiotics. Moreover, some lytic bacteriophages, which are suitable for phage therapy, are known to coexist with their host bacteria [44]. The results obtained in the present study on the Kayvirus–phage NZPT-SA7 might confirm this suggestion, since a valuable residual *S. aureus* colonization could be observed after application of the phage as a single antibacterial substance in the coated samples (Figure 3).

With regard to the study on *S. aureus*, given the sensitivity of the strain used against both vancomycin and gentamicin, the results of this work should be seen as an assessment of the synergism of the bacteriophage and the mixture of the two antibiotics. A recent study showed a reduction in phage K susceptibility to the vancomycin intermediate *S. aureus* under antibiotic exposure [45]. Our results suggest that the combination of vancomycin, gentamicin, and the K-virus representative used at a low MOI may lead to a reduction in the phage efficacy, even for vancomycin/gentamicin-sensitive *S. aureus* in planktonic culture (Figure 2). Therefore, an appropriate antibiotic–phage combination in an adequate concentration must be defined.

Our results showed that fibrin glue is a suitable drug carrier for both phages and antibiotics. However, the use of fibrin glue alone can enhance bacterial growth, which has also been reported in earlier studies [46].

It is not difficult to assume that the complexity of the phage–antibiotic system also depends on the number of simultaneously applied active ingredients. When several antibiotics and several bacteriophages are used together, different scenarios of their interaction can be expected. Therefore, further extensive studies of the most clinically relevant combinations of bacteriophages and antibiotics are promising and will lead to an improvement in the methods employed for the evaluation of phage–antibiotic synergy.

## 4. Materials and Methods

### 4.1. Materials

#### 4.1.1. Antibiotic Agents

We used a combination of vancomycin (Hikma Farmaceutica S.A., Terrugem, Portugal) and gentamicin (Ratiopharm, Ulm, Germany) solution in sterile medical-grade 0.9% NaCl, with the final concentrations of 4 mg/mL and 1.2 mg/mL, respectively, as previously described [2]. The solution was prepared immediately before use under sterile conditions.

#### 4.1.2. Bacterial Strains

In the present study, the following bacterial strains with multisensitive antibiotic susceptibility profiles (Supplementary Materials, Table S6) were used: *Staphylococcus aureus* ATCC19685 and *Escherichia coli* K12. The predetermined minimum inhibitory concentration (MIC) of gentamicin for *E. coli* K12 was <4 µg/mL, and for *S. aureus* ATCC19685, the vancomycin and gentamicin MICs were <2 µg/mL and <4 µg/mL, respectively. The bacterial broth cultures were stored in a 25% glycerol solution at −80 °C. The stock cultures were reactivated via overnight cultivation on tryptic soy agar (Oxoid, Hampshire, UK) at +37 °C, followed by overnight cultivation in tryptic soy broth (TSB) (Oxoid, Hampshire, UK) at +37 °C. The resulting second generation of the bacteria was adjusted to an optical density of 600 nm (OD_600_) and equalized to 0.2 by dilution with TSB medium. The obtained bacterial suspensions were tested for their colony-forming unit (CFUs) counts and used for the experimental procedures within 1 h. The suspensions of *S. aureus* ATCC19685 and *E. coli* K12 had concentrations of 1.6 × 10^8^ CFU/mL and 1.7 × 10^8^ CFU/mL, respectively.

#### 4.1.3. Phage Strains

We used well-characterized, strictly lytic phages. *Escherichia phage* ECD7 (Genbank accession no. KY683735.1) was used against *E. coli* [18,19]. For the control of *S. aureus*, the newly isolated *Staphylococcus phage* NZPT-SA7 belonging to the *Kayvirus* genus was used (Supplementary Materials, Table S6 and Figure S6). The phages were prepared as described previously [7], using cultivations in Roux flasks followed by phage lysate purification and the control of sterility and titer. The resulting phage suspensions in 0.9% NaCl were used at a concentration of 1 × 10^10^ plaque-forming units (pfu) per ml. The phage titer was controlled one day before use.

#### 4.1.4. Vascular Graft

Gelatin-sealed woven polyester grafts (Gelweave™, Vascutek Terumo, Inchinnan, Renfrewshire, Scotland UK) were processed into 0.5 × 0.5 cm pieces under aseptic conditions.

#### 4.1.5. Fibrin Glue

Ready-to-use surgical fibrin sealant TISSEEL (Baxter International Inc., Deerfield, IL, USA) was prepared as described previously [15]. In brief, both components of the fibrin sealant were defrosted, followed by the substitution of half of the thrombin solution with a phage suspension and gentle mixing by the inverting of the sealant syringe.

### 4.2. Methods

#### 4.2.1. Preparation of Working Antibacterial Solutions

**Figure 11:**
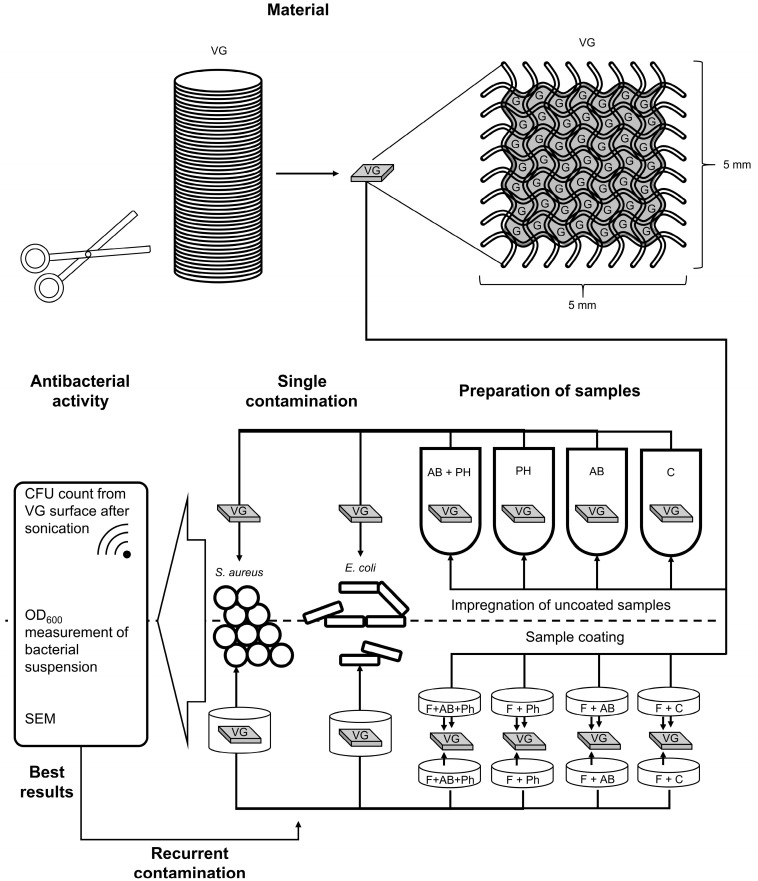
Experimental pipeline of the study. Abbreviations: VG—vascular graft; G—gelatin; F—fibrin glue; AB—antibiotics; Ph—bacteriophages; C—Control.

The presented scheme (Figure 11) outlines the experimental procedures of the study. Working solutions of the phages and antibiotics were prepared in equal concentrations for both the coated and uncoated samples. Sterile medical grade 0.9% NaCl was used in all the groups as a diluent where applicable. The mixture of antibiotic agents, as described in Section 4.1.1, was taken at a volume of 9 mL and mixed with 1 mL of a phage suspension, as described in Section 4.1.3, for the experimental groups containing both antibiotics and phages. The antibiotics or phage suspensions were substituted with 9 mL or 1 mL of sterile medical grade 0.9% NaCl for the experimental groups, which only contained phages or antibiotics, respectively. The resulting final concentrations of antibacterial agents in the working solutions were as follows: vancomycin 3.6 mg/mL; gentamicin 1.08 mg/mL; and bacteriophages 1 × 10^9^ pfu/mL. Suspensions of phages NZPT-SA7 and ECD7 were used separately for the *S. aureus* and *E. coli* groups, respectively.

#### 4.2.2. Impregnation of Uncoated Samples

The impregnation of the graft samples was performed as described previously by Ruemke et al., with the addition of a bacteriophage group [2]. Shortly afterwards, the graft materials were bathed in an antibacterial solution for 20 min at room temperature. The amount of antibacterial solution uptake was determined for additional experimental groups of graft pieces (*n* = 5) by weighing the pieces before and after impregnation. The titer of the bacteriophage uptake for these additional uncoated groups was determined using the conventional double agar overlay method, after the incubation of the impregnated samples in 4 mL of sterile TSB medium at +37 °C for 1 h [47]. The Gelweave™ grafts had a ribbed texture. In order to estimate the total surface of each sample, a series of five separate graft pieces were straightened out by pressing with the transparent lid of a 48-well plate and then measured with a caliper. Taking into account the thickness of each sample, the resulting surface area was calculated and made proportional to the weight of the sample (Supplementary Materials, Table S7). The resulting coefficient was equal to 8818 ± 211 mm^2^/g and was used to convert the weight of the graft pieces to their surface area.

#### 4.2.3. Preparation of Coated Samples

The components of the fibrin glue were transferred separately to sterile cryovials. Half of the thrombin volume was substituted with either 0.9% NaCl or antibacterial agents. The fibrin solution remained unmodified.

**Figure 12:**
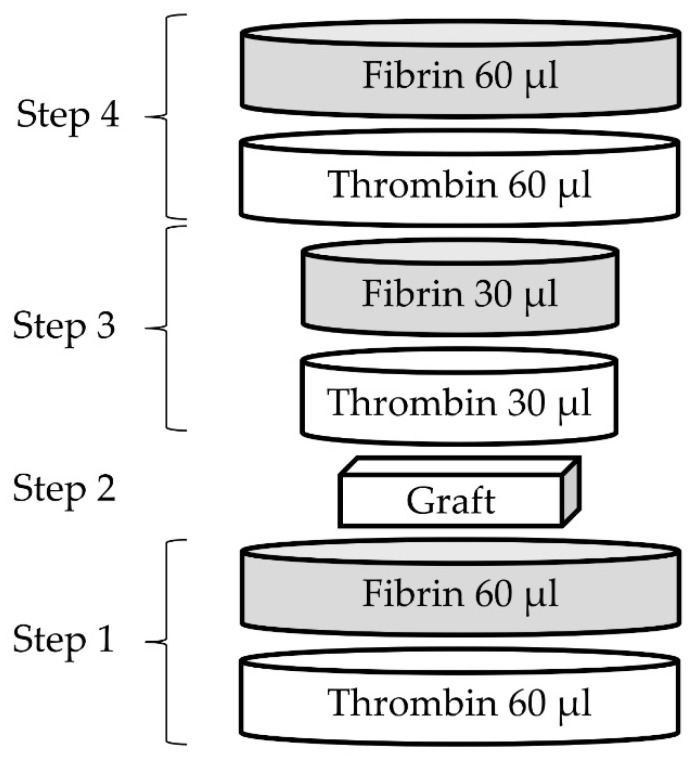
Pipeline of the sample coating.

The coating of the graft pieces was performed in sterile 48-well flat-bottom plates (Sarstedt AG, Nümbrecht, Germany) according to the following scheme (Figure 12). During the first step, 60 µL of the prepared thrombin solution was pipetted onto the bottom of the well, followed by the pipetting of 60 µL of fibrin solution. The resulting first layer of the coating was incubated at room temperature for 5 min in order to achieve a solid scaffold. In the second step, a graft sample was placed on top of the first layer. Subsequently, the third step was performed in a similar manner to the first in order to impregnate the graft sample. After another 5 min of scaffold polymerization, the next layer (step four) was added on top in the same manner. The resulting volume of the fibrin glue was 300 µL per sample and contained 75 µL of an antibacterial working solution (Supplementary Materials, Table S1). The surface area of the coated samples was estimated on a series of five separately coated graft pieces and calculated as the surface area of a cylinder. The diameter (12 mm) and height (1.5 mm) of the samples were measured with a caliper, and the resulting surface area was equal to 2.83 cm^2^. Additionally, fibrin-coated samples containing the phages NZPT-SA7 (*n* = 1) and ECD7 (*n* = 1) were prepared in order to confirm the phage release. Therefore, the samples were incubated in 4 mL of sterile TSB medium at +37 °C for 1 h, followed by phage titer assessment using the conventional double agar overlay method.

#### 4.2.4. Processing of Samples—Antibacterial Activity against Single Contamination

**Figure 13:**
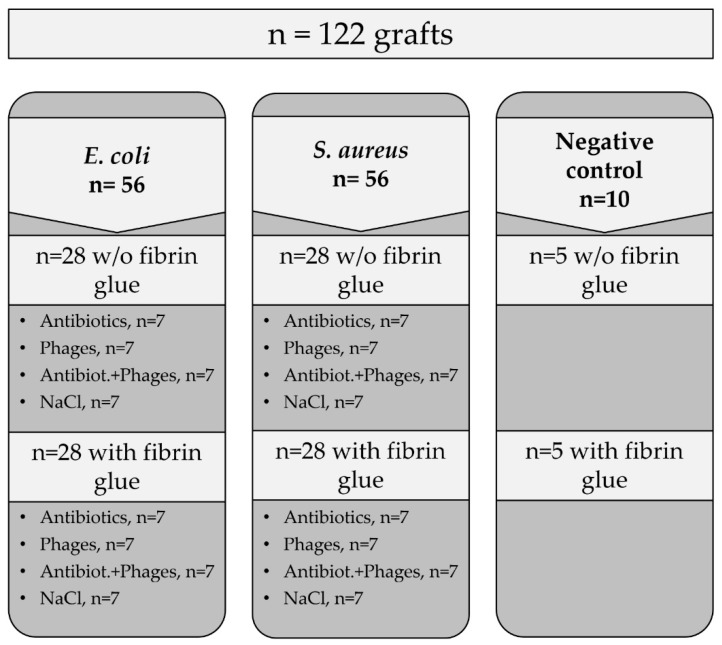
Schematic overview of experimental groups for the short-term antibacterial activity tests (CFU and OD_600_).

The further contamination of the prepared samples and the microbiological assessment were performed as we described previously [2]. In general, the samples of both the impregnated and the coated types were placed into sterile 35 mm Petri dishes (Greiner Bio-One International GmbH, Kremsmünster, Austria) and inoculated with 4 mL of a bacterial suspension of a tested bacterial strain in TSB medium. The samples were incubated at 37 °C for 24 h, followed by the measurement of the OD_600_ of the liquid part. Aliquots of 400 µL in disposable semi-micro cuvettes with a path length of 10 mm (Sarstedt AG, Nümbrecht, Germany) were tested using a BioPhotometer D30 device (Eppendorf SE, Hamburg Germany). Afterwards, all the grafts were separately transferred to 5 mL of sterile phosphate buffered saline (PBS) at pH = 7.4 in 15 mL falcon tubes; then, they underwent sonication (Elma, Singen, Germany) for 20 min at 37 °C, 40 kHz, and 180 Watt. Afterwards, the obtained suspensions of the released bacterial cells were serially diluted in decimal steps, followed by plating of 100 µL of cell suspension onto tryptic soy agar (Oxoid, Hampshire, UK) in 90 mm Petri dishes (Sarstedt AG, Nümbrecht, Germany). The CFU count was assessed after incubation of the plates for 24 h at 37 °C. The number of samples in the experimental groups is shown in the following scheme (Figure 13).

#### 4.2.5. Scanning Electron Microscopy

Scanning electron microscopy (SEM) was conducted in order to analyze the presence of the used bacteria on the surface of the uncoated and fibrin-coated graft samples for the assessment of the short-term antibacterial activity (additional *n* = 1 to each experimental group). In brief, samples of the grafts were washed in PBS (pH = 7.4) three times and fixed in 2.5% glutaraldehyde in PBS for 90 min. After fixation, the samples were washed sequentially once in PBS (pH = 7.4) and twice in deionized sterile water for 10 min each, followed by dehydration in a series of ethanol concentrations (25%, 50%, 75%, 90%, and 100%) for 10 min at each step. After the second dehydration step in 100% ethanol for 10 min, the samples were air-dried for 48 h. For the SEM imaging, the dry samples were fixed on SEM stubs using a conductive double-sided glue tape (Plano GmbH, Wetzlar, Germany). In order to avoid charge accumulation during SEM imaging, ACHESON silver painting (Plano GmbH, Wetzlar, Germany) was carefully applied on the perimeter of the cover slips using a brush. After being air-dried for at least 60 min, the prepared samples were sputter coated twice with gold–palladium for 15 s and observed under a high vacuum at a 10 kV accelerating high voltage and a 7 mm working distance using a scanning electron microscope S-3400N (Hitachi, Chiyoda-ku, Tokyo, Japan).

#### 4.2.6. Processing of Samples—Antibacterial Activity against Recurrent Contamination

Both the fibrin-coated and the uncoated samples of the vascular grafts were tested for prolonged antibacterial activity against *S. aureus* ATCC19685 and *E. coli* K12 (4 groups, *n* = 3). The samples were placed in 12 mL culture tubes (Sarstedt AG, Nümbrecht, Germany) and inoculated with 4 mL of a bacterial suspension of a tested bacterial strain in TSB medium. The samples were incubated at 37 °C for 24 h, followed by transfer to new culture tubes and inoculation with 4 mL of the bacterial suspension. The remaining overnight solution from the first culture tubes was visually assessed for the presence of turbidity. New bacterial inoculum and turbidity assessments were performed every day for up to five days.

#### 4.2.7. Sterility Test

We performed sterility tests according to the ISO 11737-1 standard in order to evaluate the complete eradication of the bacteria. Briefly, aliquots of the bacterial suspension in TSB medium after the OD_600_ measurement were transferred in amounts of 10 µL into three culture tubes: two with 5 mL of TSB medium and one with 10 mL of thioglycolate broth (TB). One TSB and one TB tube were incubated for 7 days at 30 °C. The remaining TSB tube was incubated for 7 days at room temperature (20–25 °C).

#### 4.2.8. Statistical Analysis

The data were analyzed using GraphPad Prism 8 (Graphpad Software Inc., San Diego, CA, USA). The results of the quantifications were presented as the arithmetic mean ± standard deviation (SD). Multiple comparisons between groups of samples for the antibacterial activity against single contamination and for the phage release investigation were performed using two-way ANOVA tests with Bonferroni corrections at 95% confidence intervals (CI). Multiple comparisons between groups of samples for the assessment of antibacterial substance uptake were performed using the Kruskal–Wallis test with Dunn’s correction. For the assessment of the samples during the antibacterial activity against recurrent contamination, we used the Mann–Whitney nonparametric test with a one-sided *p* value. Statistically significant differences in all the tests were considered at *p* < 0.05.

## 5. Conclusions

In our in vitro study, the bacteriophages presented themselves as an effective antibacterial tool, both alone and in combination with antibiotics. Nevertheless, for a successful use of combinations of phages and antibiotics, the previous testing of the targeted bacteria is mandatory. This may be a major hindrance for the regular clinical use in standard surgical procedures, but for the cases in which bacteria can be identified prior to the operation, targeted phage therapy remains a valuable additional option. In summary, the combination of bacteriophages, antibiotics, and fibrin glue proved to be a suitable tool for prolonging antibacterial treatment of vascular graft infections in vitro. Further studies, including in vivo trials, are needed to prove this concept with different phage–antibiotic combinations.

## Data Availability

Data is contained within the article or Supplementary Material.

## Supplementary Materials

The following supporting information can be downloaded at: https://www.mdpi.com/article/10.3390/ph16050744/s1. Table S1. Calculated antibacterial substances uptake of uncoated and coated samples; Table S2. Calculated antibiotics concentrations eluted from uncoated graft samples; Table S3. Estimation of MOI for calculated and measured (after 1 hour of incubation) phage uptake of uncoated graft samples; Table S4. Days (times) to positivity after repetitive contamination with bacterial suspensions of samples with combination of antibiotics and phages; Table S5. Antimicrobial susceptibility testing (disk diffusion method) with interpretation according to EUCAST Clinical Breakpoint Tables Version 13.0; Table S6. Genomic features of *Staphylococcus phage* NZPT-SA7*; Table S7. Calculation of surface area to weight of vascular graft pieces; Figure S1. Volume of antibacterial substances uptake of uncoated samples (each group, *n* = 5): (b) per weight of graft material; Figure S2. Calculated and measured (after 1 h of incubation) phage concentrations from uncoated graft samples (*n* = 5); * *p* < 0.05; *** *p* < 0.001; **** *p* < 0.0001; Figure S3. Fibrin glue coated vascular graft sample; Figure S4. SEM image of a graft sample with antibiotics and phages with fibrin coating after co-incubation with *E. coli.* Magnification ×55; Figure S5. SEM image of a control graft sample with fibrin coating after co-incubation with *E. coli.* Magnification ×50; Figure S6. Genomic map of *Staphylococcus phage* NZPT-SA7.
