# Supplementary material for: Combination of Bacteriophages and Antibiotics for Prevention of Vascular Graft Infections—An In Vitro Study
Source: Pharmaceuticals (Basel). 2023 May 13;16(5):744. doi: 10.3390/ph16050744 (PMC10223927; doi:10.3390/ph16050744)
Supplement: Supplementary file 1 [file pharmaceuticals-16-00744-s001.zip › pharmaceuticals-2344337-supplementary.pdf]

Supplementary materials

Combination of bacteriophages and antibiotics for prevention of vascular graft infections - an in vitro study

Stefan Ruemke [1,2,3,†,*], Evgenii Rubalskii [1,2,3,†,*], Christina Salmoukas [1,2,3], Kristina Hermes [1,2], Ruslan Natanov [1], Tim Kaufeld [1], Oleksandr Gryshkov [2,4], Vitalii Mutsenko [2,4], Maxim Rubalsky [5], Karin Burgwitz [1,2,3], Birgit Glasmacher [2,4], Axel Haverich [1,2,3], Saad Rustum [1,‡] and Christian Kuehn [1,2,3,‡]

Table S1. Calculated antibacterial substances uptake of uncoated and coated samples

	Group	Antibacterial substances uptake per vascular graft sample			
		Volume, µl†	Vancomycin, µg	Gentamicin, µg	Phage, PFU
Without fibrin	Antibiotics‡	14.60 ± 5.595	52.56 ± 20.14	15.77 ± 6.042	n.a.
	Phage NZPT-SA7‡	16.60 ± 1.949	n.a.	n.a.	$1.66\times10^{7} \pm 1.95\times10^{6}$
	Phage ECD7‡	11.20 ± 3.834	n.a.	n.a.	$1.12\times10^{7} \pm 3.83\times10^{6}$
	Antibiotics and phage NZPT-SA7‡	10.40 ± 3.050	37.44 ± 10.98	11.23 ± 3.294	$1.04\times10^{7} \pm 3.05\times10^{6}$
	Antibiotics and phage ECD7‡	13.80 ± 4.025	49.68 ± 14.49	14.90 ± 4.347	$1.38\times10^{7} \pm 4.02\times10^{6}$
With fibrin	Antibiotics‡‡	75	270	81	n.a.
	Phage NZPT-SA7‡‡	75	n.a.	n.a.	7.5×10^{7}
	Phage ECD7‡‡	75	n.a.	n.a.	7.5×10^{7}
	Antibiotics and phage NZPT-SA7‡‡	75	270	81	7.5×10^{7}
	Antibiotics and phage ECD7‡‡	75	270	81	7.5×10^{7}

†Volume of uptake (µl) was equal to the weight difference after impregnation (mg); ‡amount of antibiotics and phages in uncoated graft samples was calculated dependent on measured uptake volume during impregnation (each group n=5), data presented as mean ± SD; ‡‡amount of antibiotics and phages in coated samples was estimated dependent on used volume of an antibacterial solutions (75 µl) in a composition with other compounds of fibrin glue (in total 300 µl) per sample; n.a. – not applicable.

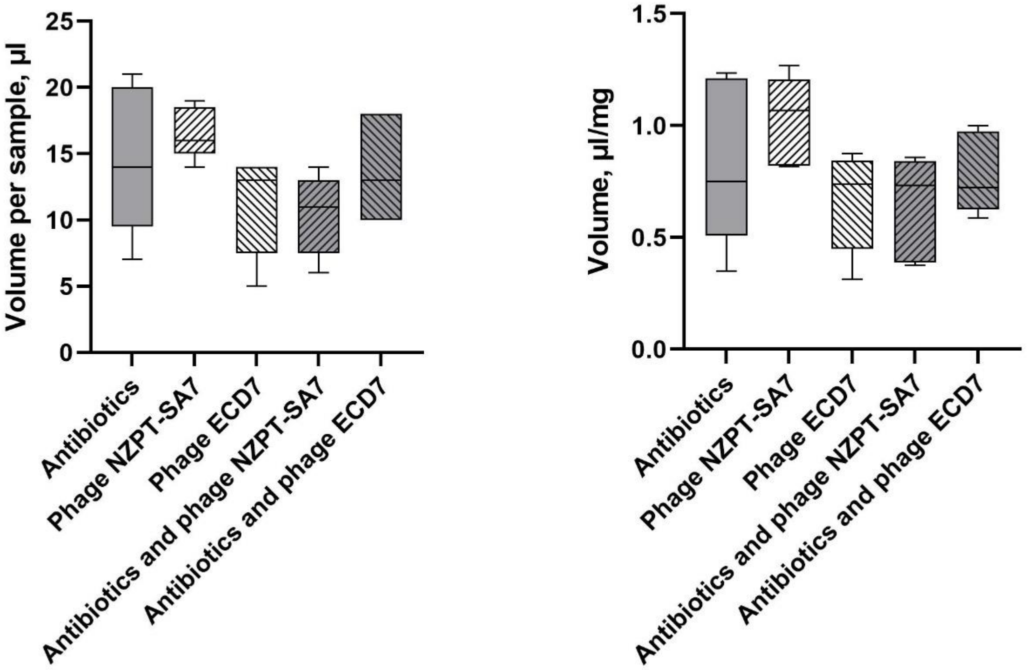

a) b)

Figure S1. Volume of antibacterial substances uptake of uncoated samples (each group, n=5): a) per graft sample; b) per weight of graft material

Table S2. Calculated antibiotics concentrations eluted from uncoated graft samples

Sample #	Antibiotics		Antibiotics and phage NZPT-SA7		Antibiotics and phage ECD7	
	Vancomycin, μg/ml	Gentamicin, μg/ml	Vancomycin, μg/ml	Gentamicin, μg/ml	Vancomycin, μg/ml	Gentamicin, μg/ml
1	12.60	3.78	10.80	3.24	16.20	4.86
2	6.30	1.89	9.90	2.97	11.70	3.51
3	18.90	5.67	12.60	3.78	9.00	2.70
4	17.10	5.13	5.40	1.62	9.00	2.70
5	10.80	3.24	8.10	2.43	16.20	4.86
**Mean**	**13.14**	**3.94**	**9.36**	**2.81**	**12.42**	**3.73**
**SD**	**5.04**	**1.51**	**2.74**	**0.82**	**3.62**	**1.09**

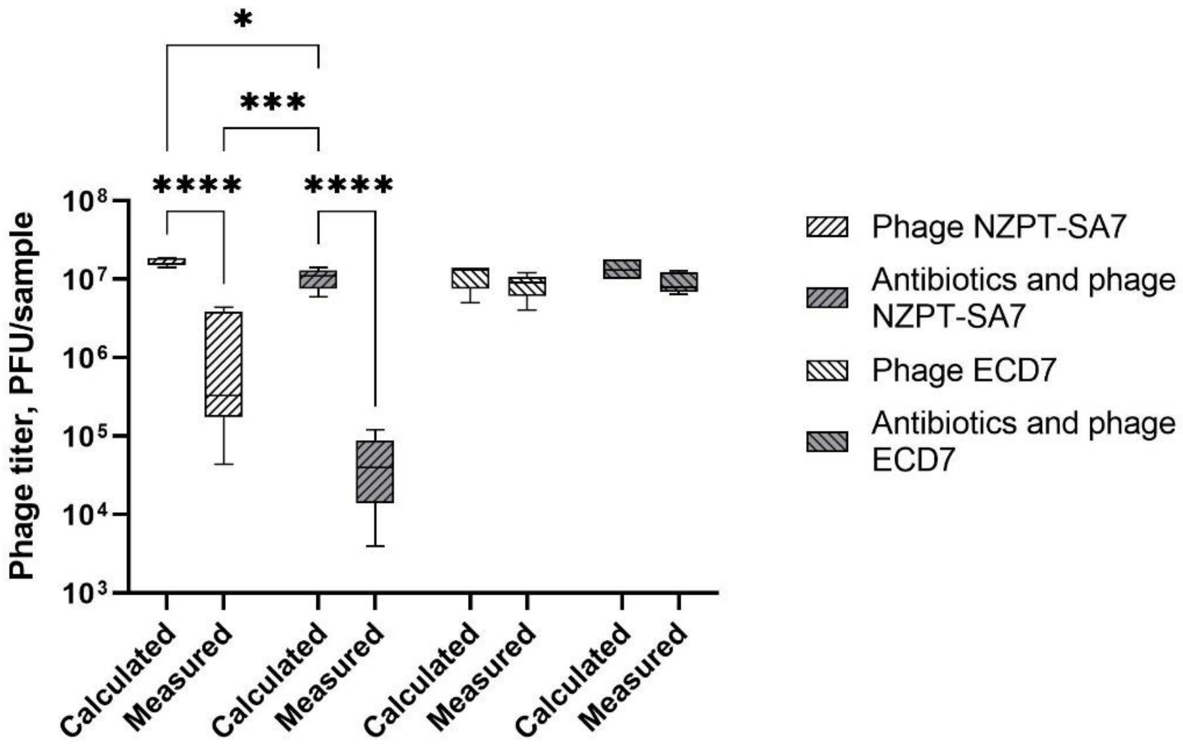

Figure S2. Calculated and measured (after 1 hour of incubation) phage concentrations from uncoated graft samples (n=5); * $p < 0.05$; *** $p < 0.001$; **** $p < 0.0001$

Table S3. Estimation of MOI for calculated and measured (after 1 hour of incubation) phage uptake of uncoated graft samples

Sample #	Phage NZPT-SA7		Phage ECD7		Antibiotics and phage NZPT-SA7		Antibiotics and phage ECD7	
	Calculated	Measured	Calculated	Measured	Calculated	Measured	Calculated	Measured
1	0.3500	0.0077	0.2353	0.2118	0.3000	0.0006	0.4235	0.2729
2	0.4500	0.1090	0.1176	0.0951	0.2750	0.0010	0.3059	0.1506
3	0.4000	0.0860	0.3294	0.2202	0.3500	0.0014	0.2353	0.1713
4	0.4750	0.0082	0.3294	0.1929	0.1500	0.0030	0.2353	0.1854
5	0.4000	0.0011	0.3059	0.2824	0.2250	0.0001	0.4235	0.3012
**Mean**	**0.4150**	**0.0424**	**0.2635**	**0.7087**	**0.2600**	**0.0012**	**0.3247**	**0.2163**

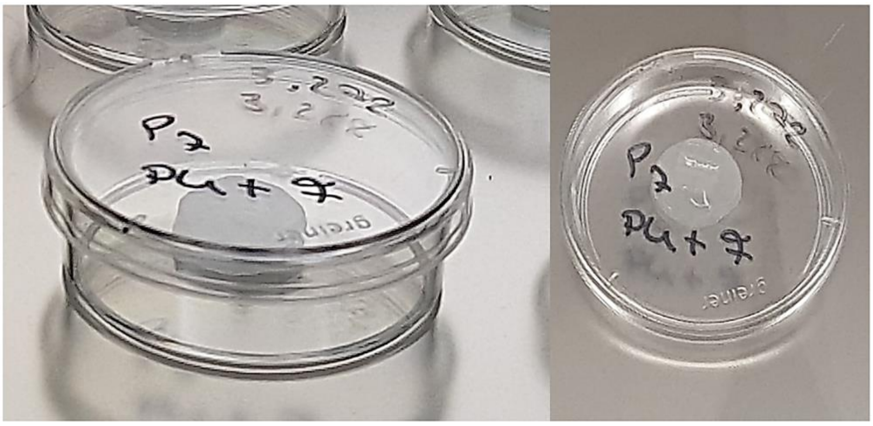

Figure S3. Fibrin glue coated vascular graft sample

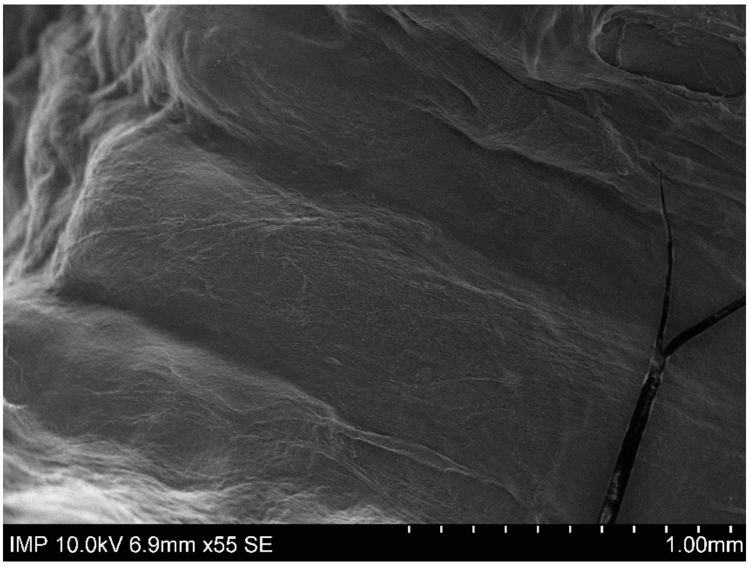

Figure S4. SEM image of a graft sample with antibiotics and phages with fibrin coating after co-incubation with *E. coli*. Magnification ×55.

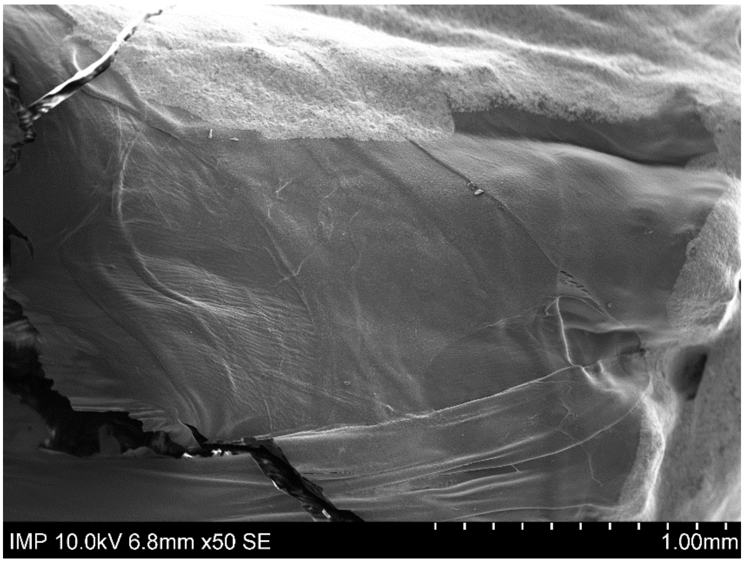

Figure S5. SEM image of a control graft sample with fibrin coating after co-incubation with *E. coli*. Magnification ×50.

Table S4. Days (times) to positivity after repetitive contamination with bacterial suspensions of samples with combination of antibiotics and phages

Days	*S. aureus*						*E. coli*					
	without fibrin glue			with fibrin glue			without fibrin glue			with fibrin glue		
	n1	n2	n3	n1	n2	n3	n1	n2	n3	n1	n2	n3
1												
2												
3												
4												
5												

White – no bacterial growth; grey – specific bacterial growth; n1-n3 – numbers of samples.

Table S5. Antimicrobial susceptibility testing (disk diffusion method) with interpretation according to EUCAST Clinical Breakpoint Tables Version 13.0

Antibiotics	Disk content (µg)	Zone diameter, mm (interpretation)	
		S. aureus ATCC19685	*E. coli* K12
Ampicillin	10	n.a.	22 (S)
Ampicillin-sulbactam	20	n.a.	25 (S)
Benzylpenicillin	1	23 (R)	n.a.
Cefotaxime	5	n.a.	34 (S)
Cefoxitin (screen only)	30	25 (S)	n.a.
Ceftazidime	10	n.a.	34 (S)
Ciprofloxacin	5	n.a.	29 (S)
Clindamycin	2	20 (R)	n.a.
Erythromycin	15	24 (S)	n.a.
Fusidic acid	10	20 (R)	n.a.
Gentamicin	10	20 (S)	19 (S)
Linezolid	10	21 (S)	n.a.
Meropenem	10	n.a.	36 (S)
Moxifloxacin	5	24 (R)	n.a.
Mupirocin	200	21 (R)	n.a.
Piperacillin	30	n.a.	28 (S)
Piperacillin-tazobactam	36	n.a.	30 (S)
Rifampicin	5	16 (R)	n.a.
Tetracycline	30	29 (S)	n.a.
Tigecycline	15	n.a.	24 (S)
Tobramycin	10	n.a.	21 (S)
Trimetoprim-sulfmethoxazole	25	22 (S)	27 (S)

S – susceptible; R – resistant; n.a. – no data.

Table S6. Genomic features of *Staphylococcus phage* NZPT-SA7*

Start, bp	Stop, bp	Feature	Description
1	7467		long terminal repeat
476	775	TreA	terminal repeat-encoded protein A similar to Staphylococcus phage MSA6
791	976	TreB	terminal repeat-encoded protein B similar to Staphylococcus phage A5W
1083	1373	TreC	terminal repeat-encoded protein C similar to Staphylococcus phage MSA6
1373	1660	TreD	terminal repeat-encoded protein D similar to Staphylococcus phage MSA6
1660	1953	TreE	terminal repeat-encoded protein E similar to Staphylococcus phage MSA6
1957	2214	TreF	terminal repeat-encoded protein F similar to Staphylococcus phage A5W
2303	2542	TreG	terminal repeat-encoded protein G similar to Staphylococcus phage MSA6
2553	2900	TreH	terminal repeat-encoded protein H similar to Staphylococcus phage A5W
3109	3447	TreI	terminal repeat-encoded protein I similar to Staphylococcus phage A5W
3758	4066	TreJ	terminal repeat-encoded protein J similar to Staphylococcus phage MSA6

Start, bp	Stop, bp	Feature	Description
4272	4556	TreK	terminal repeat-encoded protein K similar to Staphylococcus phage pSa-3
4631	4822	TreL	terminal repeat-encoded protein L similar to Staphylococcus phage A5W
5139	5627	TreM	terminal repeat-encoded protein M similar to Staphylococcus phage A5W
5795	5953	TreN	terminal repeat-encoded protein N similar to Staphylococcus phage MSA6
6023	6154	TreO	terminal repeat-encoded protein O similar to Staphylococcus phage A5W
6322	6558	TreQ	terminal repeat-encoded protein Q similar to Staphylococcus phage Staph1N
6638	7108	TreR	terminal repeat-encoded protein R similar to Staphylococcus phage A5W
7138	7263	TreT	terminal repeat-encoded protein T similar to Staphylococcus phage vB_SauM_LM12
7348	7527	TreU	terminal repeat-encoded protein U similar to Staphylococcus phage pSa-3
7861	8097	BofL	border ORF, left similar to Staphylococcus phage pSa-3
8099	8584	UboC	putative protein similar to Staphylococcus phage Fi200W
8597	9004	UboB	putative protein similar to Staphylococcus phage Fi200W
9004	9435	UboA	putative protein similar to Staphylococcus phage MSA6
9438	9629	MbpT	putative membrane protein T similar to Staphylococcus phage A5W
9626	10111	MbpA	putative membrane protein A similar to Staphylococcus phage MSA6
10104	10535	ORF26	similar to gpORF008 of Staphylococcus phage A5W
10549	11091	ORF27	putative nucleotidyl transferase similar to Staphylococcus phage JA1
11103	11591	ORF28	putative GTP cyclohydrolase II similar to Staphylococcus phage vB_SauM_LM12
11604	12002	ORF29	similar to gpORF011 of Staphylococcus phage A5W
11999	12706	ORF30	putative protein phosphatase similar to Staphylococcus phage SA5
12806	13360	ORF31	similar to gpORF013 of Staphylococcus phage A5W
13376	13693	ORF32	major tail protein
14328	14399	tRNA-Met(cat)	tRNA-Met(cat)
14679	15188	ORF33	putative non-cytoplasmic protein similar to Staphylococcus phage 812h1
15230	15448	ORF34	similar to gpORF016 of Staphylococcus phage A5W
15449	15643	ORF35	similar to gpORF017 of Staphylococcus phage A5W
15633	16370	ORF36	similar to gpORF018 of Staphylococcus phage A5W
16433	16537	ORF37	similar to gpORF019 of Staphylococcus phage A5W
16549	16788	ORF38	similar to gpORF020 of Staphylococcus phage A5W
16790	17179	ORF39	transglycosylase similar to Staphylococcus phage Team1
17278	17451	ORF40	similar to gpORF022 of Staphylococcus phage A5W
17492	17974	ORF41	hypothetical protein
18024	18566	ORF42	similar to gpORF024 of Staphylococcus phage A5W
18566	19099	ORF43	similar to gpORF025 of Staphylococcus phage A5W
19102	19266	MbpP	putative membrane protein P similar to Staphylococcus phage MSA6
19269	19544	MbpR	putative membrane protein R similar to Staphylococcus phage Fi200W
19544	20389	ORF46	putative non-cytoplasmic protein similar to Staphylococcus phage 812
20401	21519	ORF47	putative ATPase-like protein similar to Staphylococcus phage SA5
21673	21999	ORF48	similar to gpORF030 of Staphylococcus phage A5W
21992	22408	ORF49	similar to gpORF031 of Staphylococcus phage A5W
22542	22844	ORF50	putative ArpR DNA binding protein similar to Staphylococcus phage G15
22844	23032	ORF51	similar to gpORF034 of Staphylococcus phage A5W
23076	23237	ORF52	similar to gpORF035 of Staphylococcus phage A5W
23237	25285	ORF53	similar to gpORF036 of Staphylococcus phage A5W
25363	25626	ORF54	putative structural protein similar to Staphylococcus phage SA5
25643	25816	LysM	LysM domain-containing protein similar to Staphylococcus phage Sb1

Start, bp	Stop, bp	Feature	Description
25823	26401	MbpB	putative membrane protein B similar to Staphylococcus phage Stau2
26394	27020	ORF57	similar to gpORF041 of Staphylococcus phage A5W
27013	27909	Lig	putative RNA ligase similar to Staphylococcus phage Sb1
27909	28133	ORF59	putative membrane protein similar to Staphylococcus phage phiSA12
28202	28942	Phr	putative PhoH-related protein similar to Staphylococcus phage SA5
28994	29608	ORF61	hypothetical protein
29624	30049	Rbn	putative ribonuclease similar to Staphylococcus phage SA5
30039	30230	ORF63	similar to gpORF046 of Staphylococcus phage A5W
30253	30894	ORF64	similar to gpORF047 of Staphylococcus phage A5W
30884	31114	ORF65	putative transcriptional regulator similar to Staphylococcus phage G15
31117	31344	ORF66	similar to gpORF049 of Staphylococcus phage A5W
31453	32145	Tgl	putative transglycosylase similar to Staphylococcus phage A5W
32332	32967	ORF68	putative HNH endonuclease similar to Staphylococcus phage pSa-3
33034	33825	MbpS	putative membrane protein S similar to Staphylococcus phage A5W
33825	34133	MbpV	putative membrane protein V similar to Staphylococcus phage A5W
34246	34875	ORF71	putative endolysin similar to Staphylococcus phage SA5
35146	35646	ORF72	putative endonuclease similar to Staphylococcus phage SA5
35806	36609	ORF73	putative endolysin similar to Staphylococcus phage SA5
36609	37112	ORF74	putative holin similar to Staphylococcus phage Sb1
37197	37382	UphA	putative protein similar to Staphylococcus phage A5W
37544	37615	tRNA-Trp(cca)	tRNA-Trp(cca)
37622	37694	tRNA-Phe(gaa)	tRNA-Phe(gaa)
37700	37775	tRNA-Asp(gtc)	tRNA-Asp(gtc)
38929	39147	Iro	product of intergenic region ORF similar to Staphylococcus phage A5W
39625	39834	DmcB	gene B downsterminal repeat-encoded protein am of mbpC similar to Staphylococcus phage A5W
39847	40179	ORF78	putative membrane protein similar to Bacillus phage Darren
40192	40518	ORF79	putative membrane protein similar to Staphylococcus phage vB_SauM-fRuSau02
40958	41344	MbpD	putative membrane protein D similar to Staphylococcus phage A5W
41322	41600	Dmd	gene downsterminal repeat-encoded protein am of mbpD similar to Staphylococcus phage JD007
41597	42007	ORF82	similar to gpORF057 of Staphylococcus phage A5W
42022	42219	TerA	terminase large subunit similar to Staphylococcus phage Team1
42513	43484	ORF84	hypothetical protein I-MsaI similar to Staphylococcus phage MSA6
43625	45172	TerB	putative terminase similar to Staphylococcus phage MSA6
45165	45986	ORF86	putative structural protein similar to Staphylococcus phage JA1
46143	46622	ORF87	similar to gpORF060 of Staphylococcus phage A5W
46664	47851	ORF88	putative membrane-associated protein similar to Staphylococcus phage 812
47937	48278	ORF89	membrane protein similar to Staphylococcus phage pSco-10
48287	48667	ORF90	similar to gpORF063 of Staphylococcus phage A5W
48671	50362	Prt	portal protein similar to Staphylococcus phage JD007
50556	51329	Pro	putative prohead protease similar to Staphylococcus phage G15
51348	52304	ORF93	similar to gpORF066 of Staphylococcus phage A5W
52420	53811	Mcp	putative major capsid protein similar to Staphylococcus phage SA5
53903	54199	ORF95	similar to gpORF068 of Staphylococcus phage A5W
54212	55120	ORF96	putative structural protein similar to Staphylococcus phage SA5
55134	56012	ORF97	similar to gpORF070 of Staphylococcus phage A5W

Start, bp	Stop, bp	Feature	Description
56012	56632	ORF98	similar to gpORF071 of Staphylococcus phage A5W
56651	57487	ORF99	putative structural protein similar to Staphylococcus phage SA5
57489	57704	ORF100	similar to gpORF073 of Staphylococcus phage A5W
57731	59494	Tsp	putative major tail sheath protein similar to Staphylococcus phage SA5
59567	59995	ORF102	putative capsid protein similar to Staphylococcus phage G15
60092	60232	ORF103	similar to gpORF076 of Staphylococcus phage A5W
60275	60733	ORF104	hypothetical protein
60746	60940	MbpX	putative membrane protein X similar to Staphylococcus phage A5W
61022	61333	ORF106	hypothetical protein
61493	61924	ORF107	similar to gpORF079 of Staphylococcus phage A5W
61968	62504	ORF108	putative RNA polymerase similar to Staphylococcus phage G15
62560	66615	ORF109	DNA transfer protein similar to Staphylococcus phage IME-SA118
66688	69120	ORF110	putative tail lysin similar to Staphylococcus phage G15
69134	70021	ORF111	tail morphogenetic protein putative peptideglycan hydrolase similar to Staphylococcus phage phiSA12
70021	72567	ORF112	putative glycerophosphoryl diester phosphodiesterase similar to Staphylococcus phage SA5
72674	73465	ORF113	hypothetical protein similar to Staphylococcus phage phiSA12
73465	73989	ORF114	hypothetical protein similar to Staphylococcus phage phiSA12
73989	74693	bmpA	baseplate protein similar to Staphylococcus phage JD007
74708	75754	bmpB	baseplate j family protein similar to Staphylococcus phage JD007
75775	78834	TmpF	TmpF similar to Staphylococcus phage MSA6
78945	79466	ORF118	structural protein similar to Staphylococcus phage JD007
79487	82945	TmpG	TmpG similar to Staphylococcus phage MSA6
82994	83152	gpORF093	similar to gpORF093 of Staphylococcus phage A5W
83153	85075	ORF121	putative capsid and scaffold protein similar to Staphylococcus phage CH1
85098	85472	ORF122	similar to gpORF095 of Staphylococcus phage A5W
85479	86855	Rbp	receptor binding protein similar to Staphylococcus phage 812h1
86947	88695	DhlA	putative helicase similar to Staphylococcus phage SA5
88707	90320	ORF125	similar to gpORF098 of Staphylococcus phage A5W
90313	91755	DhlB	helicase similar to Staphylococcus phage JD007
91834	92871	rncA	putative exonuclease similar to Staphylococcus phage SA5
92871	93248	ORF128	hypothetical protein
93248	95167	rncB	putative exonuclease similar to Staphylococcus phage SA5
95167	95763	ORF130	hypothetical protein product similar to Staphylococcus phage G1
95778	96845	Pri	putative primase similar to Staphylococcus phage G15
96912	97250	ORF132	similar to gpORF106 of Staphylococcus phage A5W
97250	97702	ORF133	hypothetical protein
97689	98297	ORF134	putative resolvase similar to Staphylococcus phage SA5
98314	98706	ORF135	ribonucleotide reductase stimulatory protein similar to Staphylococcus phage JD007
98721	100835	ORF136	ribonucleotide reductase of class Ib (aerobic) alpha subunit similar to Staphylococcus phage IME-SA1
100849	101898	ORF137	putative ribonucleotide reductase minor subunit similar to Staphylococcus phage G15
101916	102245	ORF138	similar to gpORF112 of Staphylococcus phage A5W
102229	102549	ORF139	thioredoxin-like protein similar to Staphylococcus phage JD007
102756	103352	ORF140	similar to gpORF114 of Staphylococcus phage A5W
103362	103667	ORF141	putative integration host factor similar to Staphylococcus phage G15

Start, bp	Stop, bp	Feature	Description
103743	104615	ORF142	ORF035-like protein similar to Staphylococcus phage Sb1
104781	105293	ORF143	putative endonuclease similar to Staphylococcus phage SA5
105429	106772	PolA	DNA-directed DNA polymerase A similar to Staphylococcus phage Fi200W
107040	107747	ORF145	hypothetical protein I-KsaIII similar to Staphylococcus phage A5W
107981	108841	PolB	putative DNA polymerase similar to Staphylococcus phage SA5
108910	109152	ORF 147	similar to gpORF121 of Staphylococcus phage A5W
109169	109651	ORF 148	similar to gpORF122 of Staphylococcus phage A5W
109738	111009	ORF 149	similar to gpORF123 of Staphylococcus phage A5W
111069	111293	Rec	recombinase similar to Staphylococcus phage SAM1
111638	112606	ORF151	hypothetical protein I-MsaII similar to Staphylococcus phage MSA6
112754	113701	RecA	recombinase a similar to Staphylococcus phage Team1
113705	114058	ORF153	similar to gpORF125 of Staphylococcus phage A5W
114045	114707	Sig	putative sigma factor similar to Staphylococcus phage G15
114835	115467	ORF155	Ig-like protein similar to Staphylococcus phage pSco-10
115490	116002	ORF156	major tail protein similar to Staphylococcus phage Sb1
116017	116244	ORF157	putative structural protein similar to Staphylococcus phage SA5
116340	116600	ORF158	similar to gpORF130 of Staphylococcus phage A5W
116604	117359	ORF159	hypothetical protein
117352	118602	ORF160	putative DNA repair exonuclease similar to Staphylococcus phage SA5
118616	118984	ORF161	putative membrane protein similar to Staphylococcus phage G15
118971	119282	ORF162	similar to gpORF134 of Staphylococcus phage A5W
119346	119882	ORF163	similar to gpORF135 of Staphylococcus phage A5W
119875	120642	ORF164	sce7726 family protein similar to Staphylococcus phage G1
120620	121066	ORF165	similar to gpORF137 of Staphylococcus phage A5W
121066	121929	ORF166	similar to gpORF138 of Staphylococcus phage A5W
122301	123032	ORF167	similar to gpORF139 of Staphylococcus phage A5W
123050	123508	ORF168	putative structural protein similar to Staphylococcus phage SA5
123573	124016	ORF169	similar to gpORF141 of Staphylococcus phage A5W
124033	124737	ORF170	hypothetical protein
124799	125197	ORF171	putative membrane protein similar to Staphylococcus phage vB_SauM_LM12
125344	125559	ORF172	similar to gpORF144 of Staphylococcus phage A5W
125591	125755	ORF173	similar to gpORF145 of Staphylococcus phage A5W
125957	126133	ORF174	unnamed protein product similar to Staphylococcus phage G1
126123	126656	MbpJ	putative membrane protein J similar to Staphylococcus phage A5W
126671	126919	MbpY	putative membrane protein Y similar to Staphylococcus phage Staph1N
126931	127107	ORF177	similar to gpORF149 of Staphylococcus phage A5W
127100	127396	ORF178	similar to gpORF150 of Staphylococcus phage A5W
127444	127626	MbpK	putative membrane protein K similar to Staphylococcus phage A5W
127639	128007	ORF180	similar to gpORF152 of Staphylococcus phage A5W
128020	128367	ORF181	similar to gpORF153 of Staphylococcus phage A5W
128367	128645	ORF182	putative membrane protein similar to Staphylococcus phage vB_SauM-515A1
128715	129020	ORF183	similar to gpORF155 of Staphylococcus phage A5W
129035	129385	ORF184	similar to gpORF156 of Staphylococcus phage A5W
129385	129987	ORF185	similar to gpORF157 of Staphylococcus phage A5W
130001	130180	ORF186	similar to ORF237 of Staphylococcus phage G1
130407	130808	MbpM	putative membrane protein M similar to Staphylococcus phage A5W

Start, bp	Stop, bp	Feature	Description
130980	131105	ORF188	similar to gpORF160 of Staphylococcus phage A5W
131122	131409	ORF189	membrane protein similar to Staphylococcus phage JD007
131420	131536	ORF190	similar to gpORF162 of Staphylococcus phage A5W
131526	131789	ORF191	similar to gpORF163 of Staphylococcus phage A5W
131866	132045	MbpZ	putative membrane protein Z similar to Staphylococcus phage A5W
132060	132323	ORF193	similar to gpORF164 of Staphylococcus phage A5W
132326	132643	ORF194	similar to gpORF165 of Staphylococcus phage A5W
132644	133324	ORF195	similar to gpORF166 of Staphylococcus phage A5W
133413	133571	MbpN	putative membrane protein N similar to Staphylococcus phage A5W
133606	133806	ORF197	similar to gpORF168 of Staphylococcus phage A5W
133807	134097	ORF198	putative membrane protein similar to Staphylococcus phage phiSA12
134189	134497	ORF199	similar to gpORF170 of Staphylococcus phage A5W
134494	135402	ORF200	putative ribose phosphate pyrophosphokinase similar to Staphylococcus phage SA5
135420	136889	ORF201	putative nicotinamide phosphoribosyl transferase similar to Staphylococcus phage SA5
136968	137213	ORF202	similar to gpORF173 of Staphylococcus phage A5W
137233	137625	ORF203	similar to gpORF174 of Staphylococcus phage A5W
137627	137848	ORF204	similar to gpORF175 of Staphylococcus phage A5W
137914	138225	ORF205	similar to gpORF176 of Staphylococcus phage A5W
138228	138737	ORF206	similar to gpORF177 of Staphylococcus phage A5W
138739	139068	ORF207	similar to gpORF178 of Staphylococcus phage A5W
139074	139268	ORF208	similar to gpORF179 of Staphylococcus phage A5W
139292	139606	ORF209	similar to gpORF180 of Staphylococcus phage A5W
139621	139788	ORF210	hypothetical protein similar to ORF225 of Staphylococcus phage G1
139825	139926	BofR	border ORF, right similar to Staphylococcus phage A5W
140119	147585		long terminal repeat
140594	140893	TreA	terminal repeat-encoded protein A similar to Staphylococcus phage MSA6
140909	141094	TreB	terminal repeat-encoded protein B similar to Staphylococcus phage A5W
141201	141491	TreC	terminal repeat-encoded protein C similar to Staphylococcus phage MSA6
141491	141778	TreD	terminal repeat-encoded protein D similar to Staphylococcus phage MSA6
141778	142071	TreE	terminal repeat-encoded protein E similar to Staphylococcus phage MSA6
142075	142332	TreF	terminal repeat-encoded protein F similar to Staphylococcus phage A5W
142421	142660	TreG	terminal repeat-encoded protein G similar to Staphylococcus phage MSA6
142671	143018	TreH	terminal repeat-encoded protein H similar to Staphylococcus phage A5W
143227	143565	TreI	terminal repeat-encoded protein I similar to Staphylococcus phage A5W
143876	144184	TreJ	terminal repeat-encoded protein J similar to Staphylococcus phage MSA6
144390	144674	TreK	terminal repeat-encoded protein similar to Staphylococcus phage pSa-3
144749	144940	TreL	terminal repeat-encoded protein L similar to Staphylococcus phage A5W
145257	145745	TreM	terminal repeat-encoded protein M similar to Staphylococcus phage A5W
145913	146071	TreN	terminal repeat-encoded protein N similar to Staphylococcus phage MSA6
146141	146272	TreO	terminal repeat-encoded protein O similar to Staphylococcus phage A5W
146440	146676	TreQ	terminal repeat-encoded protein Q similar to Staphylococcus phage Staph1N
146756	147226	TreR	terminal repeat-encoded protein R similar to Staphylococcus phage A5W
147256	147381	TreT	terminal repeat-encoded protein T similar to Staphylococcus phage vB_SauM_LM12

*Function of ORFs was predicted with phage relevant sequences from the GenBank Protein database. Search request: txid28883[Organism:exp] NOT "hypothetical protein". Date: 05 May 2019.

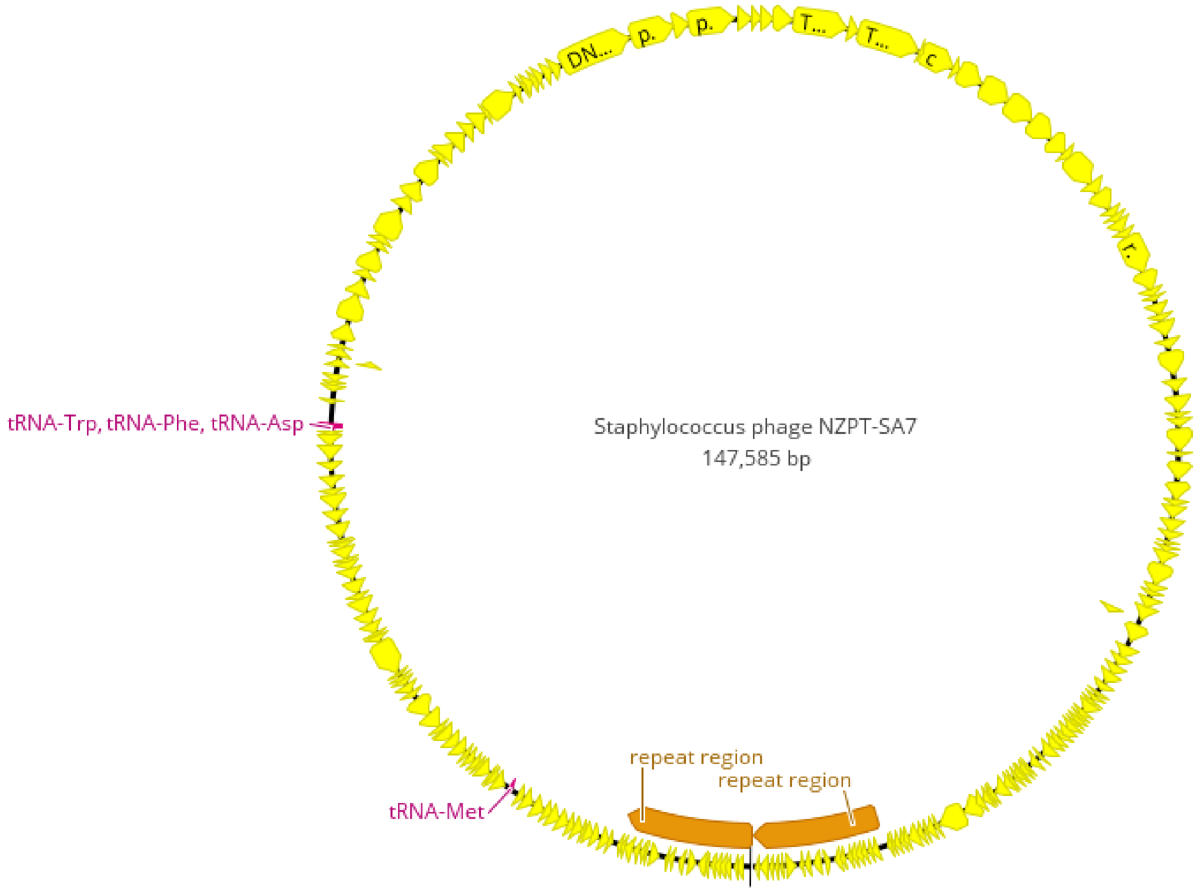

Figure S6. Genomic map of *Staphylococcus phage* NZPT-SA7

Table S7. Calculation of surface area to weight of vascular graft pieces

Sample #	Length, mm	Width, mm	Height, mm	Surface, mm²	Weight, g	Coefficient, mm²/g
1	12.0	5.5	0.2	139.00	0.0160	8687.50
2	11.8	6.6	0.2	163.12	0.0185	8817.30
3	11.8	6.4	0.2	158.32	0.0179	8844.69
4	11.9	6.3	0.2	157.22	0.0183	8591.26
5	11.7	5.8	0.2	142.72	0.0156	9148.72
					**Mean**	**8817.89**
					**SD**	**211.26**
